# Exploratory laparotomy during the battle of Mosul, 2016–2017: results from a tertiary civilian hospital in Erbil, Iraqi Kurdistan

**DOI:** 10.1186/s12873-023-00882-y

**Published:** 2023-09-23

**Authors:** Måns Muhrbeck, Aron Egelko, Rawand Musheer Haweizy, Johan von Schreeb, Andreas Älgå

**Affiliations:** 1 Department of Surgery in Norrköping and Department of Biomedical and Clinical Sciences, Linköping University, Linköping, Sweden; 2 Department of Global Public Health, Karolinska Institutet, Stockholm, Sweden; 3 College of Medicine, Hawler Medical University, Erbil, Iraq; 4 Department of Clinical Science and Education, Södersjukhuset, Karolinska Institutet, Stockholm, Sweden

**Keywords:** Armed conflict, Exploratory Laparotomy, Referral Pathways, Iraq

## Abstract

**Background:**

The Battle of Mosul (2016–2017) was an urban conflict resulting in over 9000 civilian deaths. Emergency Management Centre (EMC), located 90 km from Mosul, was designated as a civilian-run trauma centre as part of the novel Mosul Trauma Pathway. Patients necessitating exploratory laparotomy (ex-lap) provide a unique window into the system of care delivery in conflicts, given the importance of timely, resource-intensive care. However, there is insufficient knowledge regarding the presentation and outcomes for conflict-related ex-lap in civilian institutions.

**Methods:**

This is a descriptive study retrospectively analyzing routinely collected data for all patients who underwent ex-lap at EMC for injuries sustained during the battle of Mosul. Differences in demographics, pre-hospital/hospital course, and New Injury Severity Scores (NISS) were analysed using student t-test, Hotelling T-squared, and linear regression.

**Results:**

During the battle, 1832 patients with conflict-related injuries were admitted to EMC. Some 73/1832 (4.0%) underwent ex-lap, of whom 22/73 (30.1%) were children and 40/73 (54.8%) were non-combatant adults*.* Men constituted 51/73 (69%) patients. Gunshot wounds caused 19/73 (26.0%) injuries, while ordnances caused 52/73 (71.2%). Information regarding hospital course was available for 47/73 (64.4%) patients. Children had prolonged time from injury to first laparotomy compared to adults (600 vs 208 min, *p* < 0.05). Median LOS was 6 days (IQR 4–9.5); however, 11/47 (23%) patients left against medical advice. Post-operative complications occurred in 11/47 (23.4%) patients; 6/11 (54.5%) were surgical site infections. There were 12 (25.5%) patients who underwent relaparotomies after index surgery elsewhere; 10/12 (83.3%) were for failed repairs or missed injuries. Median NISS was 18 (IQR 12–27). NISS were significantly higher for women (vs men; 28.5 vs 19.8), children (vs adults; 28.8 vs 20), and relaparotomy patients (vs primary laparotomy patients; 32.0 vs 19.0). Some 3 patients died, 2 of whom were relaparotomies.

**Conclusion:**

At this civilian tertiary trauma centre, conflict-related exploratory laparotomies were associated with low morbidity and mortality. Long transport times, high rates of repeat laparotomies, and high numbers of patients leaving against medical advice raise questions regarding continuity of care along the Mosul Trauma Pathway.

**Trial registration:**

The study protocol was registered at Clinicaltrails.gov, ID NCT03490305, prior to collection of data.

**Supplementary Information:**

The online version contains supplementary material available at 10.1186/s12873-023-00882-y.

## Background

Abdominal injuries constitute approximately 10% of conflict-related injuries, with a global overall mortality of 40%, and a mortality in conflict-affiliated hospitals ranging from 5–25% [1]. Severe penetrating abdominal injuries generally require exploratory laparotomy (ex-lap) to diagnose and allow for repair of injured structures [1, 2]. Patient outcomes following conflict-related ex-laps have mostly been studied in military hospitals associated with countries affiliated to North Atlantic Treaty Organization (NATO) [3, 4]. The medical personnel and the vast majority of patients in these studies are therefore combatants.

As the theatre of war shifts to the civilian center, civilians continue to face significant burdens in accessing healthcare in conflict zones. Generally, civilians do not carry ballistic protective equipment, are excluded from advanced military healthcare systems, and face worsening resource shortages as public healthcare systems are reorganized to treat injured combatants [5–7]. In addition, armed conflicts, intrastate in particular, lead to disruptions in infrastructure and transportation, which can cause major security risks and delays for civilians to reach medical care [7]. Civilians in conflict zones therefore face both increased risk of injury and myriad obstacles to care once injured. Women and children are as well particular vulnerable within conflict zones, and face additional barriers to care given preexisting inequality [8]. Together these obstacles may be particularly problematic for severe abdominal injuries for which delays in treatment are associated with significantly increased morbidity and mortality [9].

Patients necessitating ex-lap, therefore, provide a unique window into the system of care delivery in conflicts, given the importance of timely, resource-intensive care. However, there is insufficient knowledge regarding the presentation and outcomes for conflict-related ex-lap in civilian institutions. By addressing this knowledge gap, we hope to provide information that may be used to optimize the continuum of surgical care for civilians in conflicts.

### Aim of study

To describe injury mechanisms, procedures done, and outcomes for patients who underwent exploratory laparotomy at a civilian tertiary trauma care facility in Iraq from October 17, 2016–July 20, 2017.

## Methods

### Study setting

The Battle of Mosul (October 17, 2016–July 20, 2017) was characterized by asymmetric warfare in which the Islamic State Group (IS) combined conventional warfare with newer tactics, such as human shields, improvised explosive devices (IED), and suicide bombers resulting in large numbers of civilian casualties [6, 10]. In response to the clear gaps in the trauma care for civilians, non-governmental organizations (NGOs) supported by the World Health Organization (WHO) and the Iraqi Ministry of Health (MoH) facilitated the establishment of a novel trauma system inspired by NATO and other military systems [7]. Three levels of care were set up: Trauma stabilization points (TSPs) at the front lines run by NGOs or the Iraqi MoH [11], field hospitals within an hour from the frontline, and tertiary hospitals for more complex injuries [7].

Emergency Management Centre (EMC) is located in Erbil, the capital of Iraqi Kurdistan. EMC is located 84 kms east of the city of Mosul. It was one of two tertiary hospitals designated to care for those with severe abdominal injuries during the Battle, and was assigned by the Governate to exclusively provide care to those injured during the conflict in and around Mosul [7]. Care at EMC was free of charge for patients with conflicted-related injuries. At the time of the study, EMC was a 108-bed referral hospital with 3 main ORs, 6 ICU beds, and diagnostic capabilities including computed tomography scanners. The hospital had 166 medical personnel and provided general, orthopaedic, urologic, and vascular surgery. EMC followed evidence-based guidelines for the care of traumatically injured patients and utilized a protocol for patients arriving from other facility following laparotomy. Specifically, provider-to-provider communication was expected to discuss details of the surgery and any concerns for complications, however this was not always feasible.

### Study design

This is a retrospective descriptive study using consecutively collected clinical data from EMC. All patients who underwent ex-lap at EMC from October 17, 2016, to July 31, 2017, were included. The extension of the collection period beyond the end of the battle was to ensure that all eligible patients were identified. All patients were treated according to established local hospital protocols, including a 3-day postoperative intensive care unit (ICU) stay for all patients. Data were extracted from patient charts onto paper-based forms (Supplement 1) and included information related to patient demographics, injuries, mechanism, as well as prehospital, operative, and post-operative care including complications and follow up. Initial data input into the patient record had been done by hospital staff at the time of patient presentation. Hospital staff collected prehospital data from patients themselves, ambulance personal, or any other source of information accompanying the patient. Extracted data were validated by the hospital’s chief surgeon (RMH) and the primary researcher (MM) independently. Unclear or unknown entries were classified as missing values. Collected data were compiled into a database using Epidata entry software (The Epidata Association, Odense, Denmark).

Children were defined as 15 years old or younger in accordance with international legal definitions and previous publications in the field [12, 13]. Combatant status had been decided by the emergency room nurse at time of presentation based on the presence or absence of uniform, the presence of combat casualty card, or self-report. A non-combatant was defined as a person not belonging to any warring faction while a combatant was defined as a person belonging to either warring fraction or who was injured while participating actively in the fighting [13].

Injury mechanism was initially assigned by hospital personnel at the time of presentation based on information from ambulance service, patient, other sources of information accompanying the patient, or the appearance of the injuries and their distribution. Injury mechanism was then categorized during data extraction into three categories; gunshot wound (GSW), ordnance, or IED injury, consistent with previous research on the battle [14]. A GSW was defined as having been caused by any weapon firing nonexplosive kinetic projectiles. An ordnance injury was defined as having been caused by any conventional explosives such as hand-held or rocket-propelled grenades. An IED injury was defined as having been caused by any explosive device constructed and or deployed in a non-conventional way.

Intraabdominal injuries were recorded by the attending surgeon following laparotomy. Heart rate, systolic blood pressure, and haemoglobin concentration were categorized as within normal range or not, based on the reference values per age category as listed in the Advanced Trauma Life Support manual and the WHO definition of anaemia [15, 16]. Injury Severity Scores (ISS) and New Injury Severity Scores (NISS) were calculated based on available operative data [17]. When ambiguity existed in categorizing ISS/NISS scores, the lowest possible score was chosen. The study protocol was registered at Clinicaltrails.gov, ID NCT03490305, prior to collection of data. Ethical approval was obtained from the research ethics committee of Hawler Medical University in Erbil, ethics issue number 20/21–11-2017.

### Statistical methods

Descriptive values are given as actual numbers, percentage, and median with interquartile range, as appropriate. Categorical variables were analysed using Fischer’s exact test. Means were compared using student t-test or Hotelling T-squared tests as appropriate. Medians were compared with Mann–Whitney-U test. Linear regression was performed to analyse the impact of NISS on length of stay (LOS) given previously published associations [18]. Regression was performed in both a univariate model and a multivariate model controlling for age, sex, and whether the patient was discharged at the completion of their hospital care (as opposed to early discharge). *P*-values (two-tailed) less than 0.05 were considered significant. Statistical analysis was done using Stata software version 17 (StatCorp, College, Station TX, USA).

## Results

**Fig. 1:**
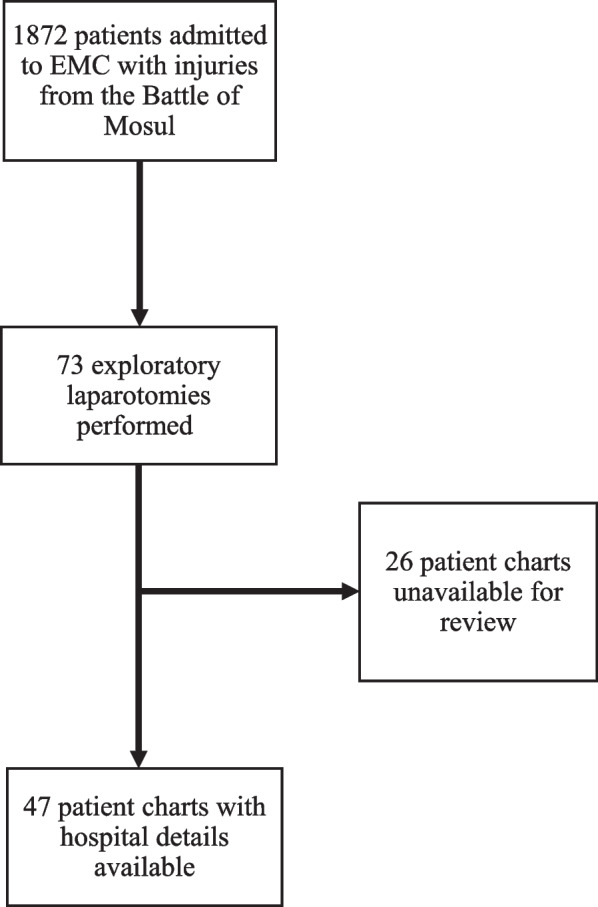
Flowchart of patient inclusion in the study

A total of 1832 patients with conflict-related injuries were admitted to EMC between October 17, 2016, and July 31, 2017. Out of these, 73/1832 (4.0%) patients underwent ex-lap (Fig. 1). Within the ex-lap cohort, 22/73 (30.1%) were children and 40/73 (54.8%) were non-combatant adults. Sex, age, injury mechanism, and combatant status for the included patients are shown in Table 1. Combatants were exclusively male; there were no significant differences in sex distribution for children and non-combatant adults. GSWs caused 19/73 (26.0%) of injuries necessitating ex-lap, while ordnances caused 52/73 (71.2%). There were no significant age, sex, or combatant status differences between the different injury mechanisms. For 26/73 (35.6%) patients, the complete chart containing details regarding hospital course was unable to be accessed at the time of the study. For these patients, only the face sheet with demographic information and injury mechanism was available.

**Table 1:** Characteristics and injury mechanisms for patients undergoing exploratory laparotomy at EMC during the Battle of Mosul

		Male	Female	*P*-value
Number of patients		51	22	
Age, median (IQR)		25.00 (13.00, 37.00)	16.50 (7.00, 35.00)	0.18
Patient Identity	Child	13 (25%)	9 (41%)	0.035
	Noncombatant	27 (53%)	13 (59%)	
	Combatant	11 (22%)	0 (0%)	
Cause of Injury	Ordnance	38 (75%)	14 (64%)	0.51
	Gunshot (GSW)	12 (24%)	7 (32%)	
	IED	1 (2%)	1 (5%)	

### Patients with complete charts available for review

Sufficient documentation existed to perform a more detailed chart review for 47/73 (64.4%) patients (Fig. 1). Age, sex, and injury mechanism were not significantly different between those with and without complete charts available for review. All patients received at least first aid at a TSP prior to arrival at EMC. Some 33/47 (70.2%) patients came directly from a TSP, rather than having been first transferred to a Field Hospital per the pathway. Preoperative imaging of any kind (x-ray, ultrasound, or CT scan) was utilized in 28/47 (59.6%) patients. Prior to admission to EMC, 12/47 (25.5%) patients underwent ex-lap elsewhere and one (2.1%) patient also underwent an above knee amputation prior to admission. There were no significant differences between children, non-combatants, and combatants in type of care received prior to admission. Time of injury was recorded for 23/47 (48.9%) patients, all of whom underwent their first operation at EMC. The median time from injury to presentation to EMC was 120 min (IQR 80–355). Children had a significantly longer time to admission compared to adults (600 vs 208 min, *p* = 0.03).

Complete vital signs were available for 28/47 (60.0%) patients at admission. There were no significant differences in terms of age, sex, combatant status, mechanism of injury, or injury severity between those with and without vital sign data recorded. Of those with vital signs available, 10/28 (35.7%) patients were tachycardic and 8/28 (28.6%) were hypotensive with no significant difference between children, non-combatants, and combatants. Baseline hemoglobin values were recorded for 39/47 (83.0%) patients. Of these, 27/39 (69.20%) patients were anaemic. Children had significantly lower haemoglobin concentration than adults upon admission (children: median 10.0 g/dl, IQR 8.2–11.2; adult: median 11.9 g/dl, IQR 11.0–13.5, *p* = 0.02).

Abdominal perforation site was equally divided between the four quadrants. Concomitant injuries were present for 36/47 (76.6%) patients and were more common among children (92.9%) and non-combatant adults (79.2%) than among combatants (44.4%) (*p* = 0.025). Concomitant injuries were most frequent on the upper extremity (10/36; 27.8%), back (8/36; 22.2%), and chest wall (7/36; 19.4%). Procedures done at ex-lap are shown in Table 2. Bowel repair (including resection with anastomosis) was the most commonly performed procedure (24/47; 51.1%) followed by removal of foreign bodies (15/47; 31.9%). Amongst those who required bowel repair, 12/24 (50.0%) had exclusively small bowel injuries, 5/24 (20.8%) had exclusively large bowel injuries, and 7/24 (29.2%) had injuries to both. Abdominal drains were placed in 34/47 (72.3%) patients and chest tube placement occurred in 11/47 (23.4%) patients. Two (4.2%) patients had negative ex-laps: one patient with ordnance injury to the posterior left chest and one patient with a GSW to the anterior lower chest, neither of whom underwent preoperative imaging. Five (10.6%) patients underwent relaparotomies following initial surgery at EMC for various reasons: bleeding, fascia rupture, suture of rectal stump, failed anastomosis, and gastric fistula. Amongst those who underwent initial surgery at EMC, there were no significant differences in procedures done when comparing children, non-combatants, and combatants.

**Table 2:** Procedures performed during exploratory laparotomy at EMC during the Battle of Mosul

Type of procedure	All patients *n* = 47 (%)^a^
Damage control	2 (4%)
Foreign body removal	15 (32%)
Primary diaphragm repair	6 (13%)
Chest tube placement	11 (23%)
Bowel repair^b^	24 (51%)
Small bowel resection^c^	11 (23%)
Colon resection^c^	2 (4%)
Stoma creation	5 (11%)
Vascular repair (any)	2 (4%)

^a^Patients may have been subjected to several procedures which results in more procedures than patients and a sum of percentages more than 100

^b^Including primary repair and resection with anastamosis

^c^All resections were followed by anastamosis

ICU stay longer than the protocolized 3 days post-surgery occurred in 16/47 (34.0%) patients, with a median prolonged stay of 4 days (IQR 4–6 days). There was no significant difference by sex, age, or combatant status for patients who required prolonged ICU stay. Post-operative complications occurred in 11/47 (23.4%) patients; of which 6/11 (54.5%) were surgical site infections. Blood products were given to 33/47 (70.2%) patients, with a median of two units (IQR 1–6). Total transfusion amounts were not associated with hemoglobin concentration on admission (*p* = 0.90).

Median LOS was six days (IQR 4–10). There were 10/47 (21.3%) patients who left the hospital against medical advice (AMA), six of whom were children (*p* = 0.02 when compared to the proportion who were adults). Transfer from EMC to another facility occurred in 8/47 (17.0%) patients, four of whom were combatants (*p* = 0.03 when compared to the proportion who were non-combatants). Overall, 22/47 (46.8%) patients left the hospital for reasons other than completion of hospital care. There were no significant differences by sex, age, or combatant status of the proportion who completed their hospital care at EMC. Amongst those who completed their care, the median LOS was 6.5 days (IQR 5–9) and there were no significant differences in LOS when comparing combatants and non-combatants, children and adults, or men and women. Three (6.4%) patients, all non-combatants, died during their hospital stay. The causes of death for these three patients were listed as: abdominal sepsis, acute renal failure, and haemorrhagic shock.

Subsequent surgeries within the same admission for wound management occurred in 25/47 (53.2%) patients. Some 22/47 (47%) patients underwent delayed primary closure, five (11%) patients underwent split-thickness skin grafting, and nine (19%) patients underwent wound debridement. Routine follow-up appointments post-discharge were scheduled for 34/47 (72.3%) patients. However, only 13/34 (38.2%) patients presented for their scheduled follow up appointment.

### Analysis of (New) Injury Severity Scores

Median ISS was 13 (IQR 9–18) and median NISS was 18 (IQR 12–27) for the 47 patients that had complete charts available for review. The mean difference between ISS and NISS for individual patients was 7.3 (*p* < 0.001).

NISS values were significantly higher among women (28.5 vs 19.8, *p* = 0.04), children (28.8 vs 20, *p* = 0.04), civilians (24.7 vs 14.6, *p* = 0.03), and those who had had prior surgery (32.0 vs 19.0, *p* = 0.002). This is shown in Table 3. In univariate analysis NISS was associated with LOS with a coefficient of 0.47 (*p* < 0.001; R^2^ = 0.26). This association was similar in a multivariate model controlling for age, sex, combatant status, and completion of hospital care, with a coefficient of 0.40 (*p* < 0.01; R^2^ = 0.27). Within this multivariate model, age, sex, and combatant status were not significantly associated with LOS. NISS scores were not associated with length of ICU stay, blood product transfusion, complication rates, need for multiple surgeries, or mortality. Table 4 shows the results of the regression models for NISS on LOS.

**Table 3:** Comparison of mean NISS scores between different patient populations

Category		NISS^a^	*P*-value
Sex			0.04
	Men	19.8	
	Women	28.5	
Age			0.04
	Adult	20	
	Child	28.8	
Combatant Status			0.03
	Combatants	14.6	
	Civilians	24.7	
Prior Surgery			0.002
	Yes	32	
	No	19	

^a^New Injury Severity Score (mean)

**Table 4:** Relationship between NISS and Hospital and ICU length of stay

	Coefficient	R2	*P*-value
Linear Regression of NISS^a^ on LOS^b^			
Univariate	0.47	0.26	< 0.001
Multivariate^d^	0.37	0.35	0.008
Linear Regression of NISS on ICU^c^ LOS			
Univariate	0.01	0.002	0.9
Multivariate^d^	-0.01	0.01	0.5

^a^New Injury Severity Score, ^b^Length of Stay, ^c^Intensive Care Unit,

^d^Controlling for Age, Sex, Combatant Status, and Completion of Hospital Care

### Sub-group analysis of patients who experienced exploratory laparotomy prior to admission

Prior to ex-lap at EMC, 12/47 (25.5%) patients had undergone laparotomies at other care facilities. Of these, 7/12 (58.3%) were male, 6/12 (50.0%) were adults, and 11/12 (91.6%) were non-combatants. One non-combatant and one combatant underwent planned relaparotomies at EMC after primary damage control surgery elsewhere. The remaining 10/12 (83.3%) patients were relaparotomies due to failed repair and/or missed injury at the primary laparotomy. Median time between primary laparotomy and relaparotomy at EMC was 5.5 days (IQR 1–7). For six of these patients, the procedures done at the primary laparotomy was not communicated to EMC. Those with prior laparotomy had longer mean ICU stay (4.5 vs 2.9, *p* < 0.01) and hospital stay (20.7 vs 7.6, *p* < 0.01) compared to those undergoing first surgery at EMC. The impact of prior laparotomy on both ICU and hospital LOS remained significant in a multivariate model controlling for age, sex, and NISS score. Of note, the number of days between index laparotomy and first surgery at EMC was not associated with NISS, LOS, rate of complications, or blood transfusion needs. Two out of the three patients who died during hospital stay underwent index laparotomy before arriving at EMC, both of whom had missed injuries (one had an intraperitoneal rectal laceration and the other a full-thickness caecal injury).

## Discussion

In this study of patients undergoing exploratory laparotomies at a civilian tertiary trauma care facility we found an overrepresentation of patients who were noncombatants, as well patients who were women or under 15, compared to the overall patient burden at the facility during the 2016–2017 Battle of Mosul [14]. This overrepresentation may represent a higher risk of abdominal injury among these groups due to factors such lack of access to protective equipment, e.g. ballistic armor (which covers the torso) and armored vehicles. Our patient demographics and survival data were similar to a study of 898 civilian patients who underwent conflict-related ex-laps in Syria [19]. The distribution of injuries and surgery performed at laparotomy were as well similar to a case series of 66 conflict-related laparotomy performed at a civilian-run hospital in Afghanistan [20].

Our study reaffirmed the utility of NISS for predicting LOS amongst injured patients, however it did not correlate to other metrics of healthcare utilization. This may be due to limitations in the retrospective data collection, or due to the protocolized nature of healthcare delivery, both of which are discussed in the limitations below. NISS scores, which are calculated based on the three most severe injuries regardless of body region, were notably higher than ISS scores, which are calculated based on the three most injured body regions. This difference between the ISS and NISS likely reflects the high burden of multiple abdominal injuries, with a comparatively low burden of serious extra-abdominal injuries in these patients. NISS scores were lower and transport times were longer in our study compared to studies of military hospitals in Iraq [21]. These findings, combined with the low mortality in our study, suggest high prehospital mortality. Additionally, the lack of concomitant injuries to major thoracic structures amongst our patients indicates that patients with serious thoracic injuries likely died prior to presentation given the rapid mortality of such injuries without immediate intervention [22]. The fact that civilians, women, and children all had higher NISS scores may indicate an increased vulnerability to injury amongst these groups, or may represent that more seriously injured combatants, men, and adults were able to access care closer to Mosul.

Multiple, intersecting barriers to safe and timely patient transport likely underly the prolonged transport times in our study. For example, transit between TSP, Field Hospitals, and Erbil often occurred in ambulances or regular vehicles which lacked adequate resources and staff [6]. Intrastate conflicts often result in major disruptions to infrastructure with myriad effects on civilian health [23]. Our results highlight the need for investment in robust transit systems during periods of conflict given the documented relationship between transport times and patient outcomes [9, 21].

The percentage of patients in our study who left AMA (20%) is far higher than reported in other contexts. The Médecins Sans Frontières hospital in Kunduz, Afghanistan reported a left AMA percentage of 2.4% amongst patients admitted with traumatic injuries [24], which is similar to a left AMA percentage of 1.8% reported amongst traumatically injured patients in the United States [25]. Reasons for leaving AMA have been previously conceptualized into two categories: patient/family related factors and healthcare related factors [26], both of which were likely present in this population. Particularly given the distance from EMC to Mosul, patients may have needed to leave to reunite with family or attend to matters in Mosul such as destruction of property due to the fighting. On the healthcare side, strict adherence to protocols which included minimum lengths of stay may have led to a lack of clarity between clinicians and patients regarding the point at which care was complete. There may as well be some element of data misclassification, with patients who failed to present for follow up being incorrectly marked as having left AMA, given that none of the patients who were marked as having left AMA presented for scheduled follow up.

The impact of the trauma pathway as suggested by the WHO is difficult to assess. Our demographic data are similar to data from all patients presenting to Mosul General Hospital, one of the field hospitals in the conflict [27, 28]. Additionally, many patients in our study were transferred directly from TSPs to EMC, thus bypassing the field hospitals. Together, these facts raise questions regarding adherence to the WHO pathway. Lack of adherence to the WHO pathway may have limited the pathway’s ability to improve patient transport times and pre-hospital mortality, although such an analysis of pathway adherence is beyond the scope of this paper. The relatively high rate of reoperation for failed repair or missed injury raises questions about the quality of surgical care at earlier points along the pathway. High rates of reoperation with poor communication between healthcare facilities have been previously described in contemporary middle east conflict [29].

### Limitations

Key amongst the limitations in this study is the overall low quality of documentation on which the analysis is based. This includes both the sparse documentation of prehospital factors, as well as the large number of clinical variables throughout the hospital course that were not documented. Poor documentation is a persistent challenge in research in conflict zones [5, 29] and likely reflects on the ground prioritisation of patient care over robust documentation.

Length of stay analysis, both ICU and overall hospital, was limited by several factors. The large number of patients who left for reasons other than the completion of their hospital care limits the utility of the metric. In particular, the Iraqi military desired to rapidly transfer their soldiers to military hospitals, as evidenced by the higher proportion of combatants transferred. However, the overall proportion of individuals who completed hospital care did not vary between groups. Per local hospital protocol all patients were assigned a 3-day ICU stay following laparotomy, and thus length of ICU stay may not accurately reflect injury severity.

The present study only included data from a single centre and is therefore not representative for all patients treated for conflict related injuries during the study period. The small number of patients with abdominal injuries requiring surgical intervention makes it difficult to generalize the results from this study. The findings in regards to patient characteristics and injury patterns are however similar to those found in previous studies of the same conflict [6, 14, 28]. Additionally, the overall rates of abdominal injury within our population are similar to reports from the International Committee of the Red Cross regarding burden of abdominal injury at their various conflict-affiliated hospitals [5]. The cross-sectional design using routinely collected data allowed for efficient data collection, however the overall data quality was low. Furthermore, the ability for follow up was limited.

There is a risk of sampling bias as we do not know how the decision was made to bring a patient to EMC as opposed to the other tertiary centre. However, given the similarity of our results to studies of other hospitals in this conflict, we do not anticipate a systemic bias impacting which sorts of patients ended up at EMC. Survival bias is likely also present given the low hospital mortality, and thus the most injured patients, or those in highest need of surgical care, may not have made it to EMC. Consequently, our results cannot be extrapolated to all conflict-related abdominal injuries, given the multiple unknown prehospital factors in our study. Additionally, there is a risk of misclassification bias along multiple parameters, however we expect this misclassification to be nondifferential and thus blunting, rather than exaggerating, intergroup differences. The analysis of injury severity scores was based on secondary analysis of documented injuries, and thus several opportunities for misclassification exist. Significant amounts of clinical data were missing, limiting analytic abilities. Given the lack of significant difference between those patients with and without available clinical data, the risk of significant selection bias impacting the clinical variables is minimal. We cannot exclude the risk of hidden bias since there might be confounders that are not known to us given the limitation of routinely collected patient data.

### Lessons learned

A large proportion of the patients that presented with penetrating abdominal injuries were children, many of whom had injuries to multiple organs. Surgical care providers in armed conflict zones must therefore possess a broad set of skills to provide the requisite care. Furthermore, we experienced prolonged transport times and lack of structured information transfer between caregivers. This lack of information about the circumstances of injury and the absence of structured communication between surgical teams across at different facilities resulted in medical approaches which didn’t directly match civilian practice in non-conflict settings. This emphasises that future civilian trauma pathways in conflicts zones should not only entail coordination, but also robust systems for transport of patients and transfer of information between care providers.

## Conclusions

At EMC, a civilian tertiary trauma centre during the Battle of Mosul, exploratory laparotomies among a largely civilian patient population were associated with low morbidity and mortality. Prolonged transport times, high rates of repeat laparotomies, and high numbers of patients leaving against medical advice raise questions regarding continuity of care along the WHO Mosul Trauma Pathway. Further research is needed on how to optimize systems to provide consistent, timely, and high-quality medical care to civilians in conflict zones.

### Supplementary Information

**Additional file 1.**

## Data Availability

The datasets generated and/or analysed during the current study are not publicly available due as they contain protected patient information but are available from the corresponding author on reasonable request.

## Abbreviations

Ex-lap: Exploratory Laparotomy
NATO: North Atlantic Treaty Organization
IS: Islamic State Group
IED: Improvised Explosive Device
NGO: Non-governmental Organizations
WHO: World Health Organization
MoH: Iraqi Ministry of Health
TSP: Trauma Stabilization Points
EMC: Emergency Management Centre
ICU: Intensive Care Unit
LOS: Length of Stay
ISS: Injury Severity Score
NISS: New Injury Severity Score
AMA: Against Medical Advice
