# Supplementary material for: Exploratory laparotomy during the battle of Mosul, 2016–2017: results from a tertiary civilian hospital in Erbil, Iraqi Kurdistan
Source: BMC Emerg Med. 2023 Sep 23;23:113. doi: 10.1186/s12873-023-00882-y (PMC10518085; doi:10.1186/s12873-023-00882-y)
Supplement: Supplementary file 1 — Additional file 1. [file 12873_2023_882_MOESM1_ESM.pdf]

Exploratory laparotomy following penetrating abdominal injuries: cohort study from a referral hospital in Erbil, Kurdistan region in Iraq. Case Report Form (CRF), version 180302

Patient Data

Patient no ☐

1. Time of admission ☐ / / (day month year) ☐ : (hours minutes) ☐ Unknown

2. Age ☐ in years ☐ Unknown

3. Sex ☐ Male ☐ Female ☐ Unknown

4. Ethnicity ☐ Arabic ☐ Kurdish ☐ Other ☐ Unknown

____

If other, please specify

5. Civilian/Combatant ☐ Civilian ☐ Combatant ☐ Unknown

If combatant ☐ Iraqi ☐ Peshmerga ☐ IS ☐ Unknown ☐ Other

____

If other, please specify

Investigators signature ______________________ / / (day month year)

Validated by and date: ______________________

EMC Emergency Managment Center سه نته ری چاره سه ری فریاكوزای Karolinska Institutet LINKÖPING UNIVERSITY

Exploratory laparotomy following penetrating abdominal injuries: cohort study from a referral hospital in Erbil, Kurdistan region in Iraq. Case Report Form (CRF), version 180302

Injury Data

Patient no [ ]

6. Time of injury

:	/ /	
hours minutes	day month year	Unknown

7. Geographical location where injury occurred

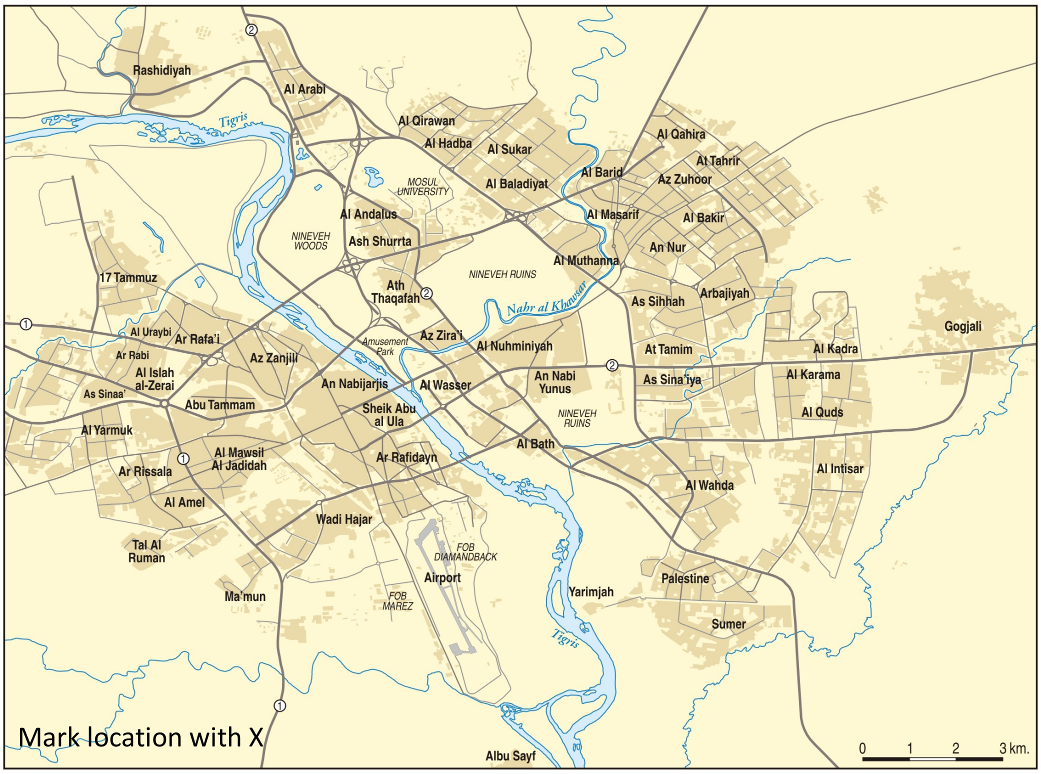

[ ]	[ ]
Unknown	Other

____

If other, please specify location where injury occurred

____

Investigators signature ____________________ / /

day month year

Validated by and date: ____________________

EMC Emergency Managment Center ‎سه نته ری چاره سه ری فریاگوزای‎ Karolinska Institutet LINKÖPING UNIVERSITY

Injury Data continued

Patient no ☐

8. War-related injury

☐ Yes ☐ No ☐ Unknown

9. Cause of injury
(If several causes number in same order as question 20 and 21)

☐ Bullet ☐ Shell ☐ Mine
☐ IED ☐ Unknown ☐ Other

If other, please specify

10. Mechanism of injury
(If several number in same order as question 20 and 21)

☐ Gunshot ☐ Stab ☐ Blast
☐ Crush ☐ Fragment ☐ Unknown
☐ Burn ☐ Other ☐ Traffic accident

If other, please specify

Investigators signature ____________________ / /
day month year

Validated by and date: ____________________

EMC Emergency Managment Center سه نته ری چاره سه ری فریاگوزای Karolinska Institutet LiU LINKÖPING UNIVERSITY

Prehospital Care Data

Patient no ☐

11. Care prior to admission to EH
(If several number in chronological order)

☐ First aid ☐ Medical ☐ None given

☐ Unknown ☐ Surgery

If surgery, please specify procedure done

___ / ___ / ___ of procedure
day month year

12. Provider of care prior to EH
(If several number in chronological order)

☐ MSF ☐ Aspen ☐ ICRC

☐ Unknown ☐ Samaritan Purse

☐ None ☐ Department of Health

Other, please specify

Investigators signature ______________________ / ___ / ___
day month year

Validated by and date: ______________________

Prehospital Care Data cont.

Patient no ☐

13. Mode of transport to EH
(If several number in chronological order)

☐ Military ☐ Private car

☐ Motorcycle ☐ Ambulance ☐ Unknown

____
Other, please specify

14. Medical history

☐ Healthy ☐ Unknown ☐ Prior surgery ☐ Prior medical

____
If prior surgery, please specify procedure(s)

____

____

____
If prior medical condition(s), please specify

____

____

Investigators signature ______________________ / /
day month year

Validated by and date: ______________________

Exploratory laparotomy following penetrating abdominal injuries: cohort study from a referral hospital in Erbil, Kurdistan region in Iraq. Case Report Form (CRF), version 180302

Clinical Data at OPD

Patient no ☐

15. Body temperature ☐ in Celsius. ☐ Unknown

16. Heart rate ☐ in beats/min ☐ Unknown

17. Blood pressure ☐ / ☐ in mmHg ☐ Unknown
Systole Diastole

18. Pregnant ☐ Yes ☐ No ☐ Unknown

19. Pregnancy status based on ☐ Lab ☐ Question ☐ Status ☐ Unknown

20. Site and extent of abdominal wound

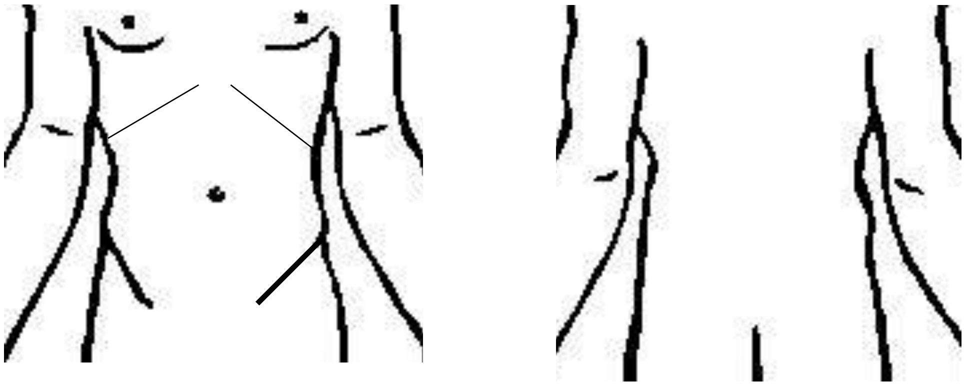

If >2 injuries use remarks

Mark each wound by number

1. ______________________________
Brief description of size and depth

2. ______________________________
Brief description of size and depth

Investigators signature ______________________ / /
day month year

Validated by and date: ______________________

EMC Emergency Managment Center; Karolinska Institutet; LiU Linköping University

Clinical Data at OPD cont.

Patient no

21. Other injuries

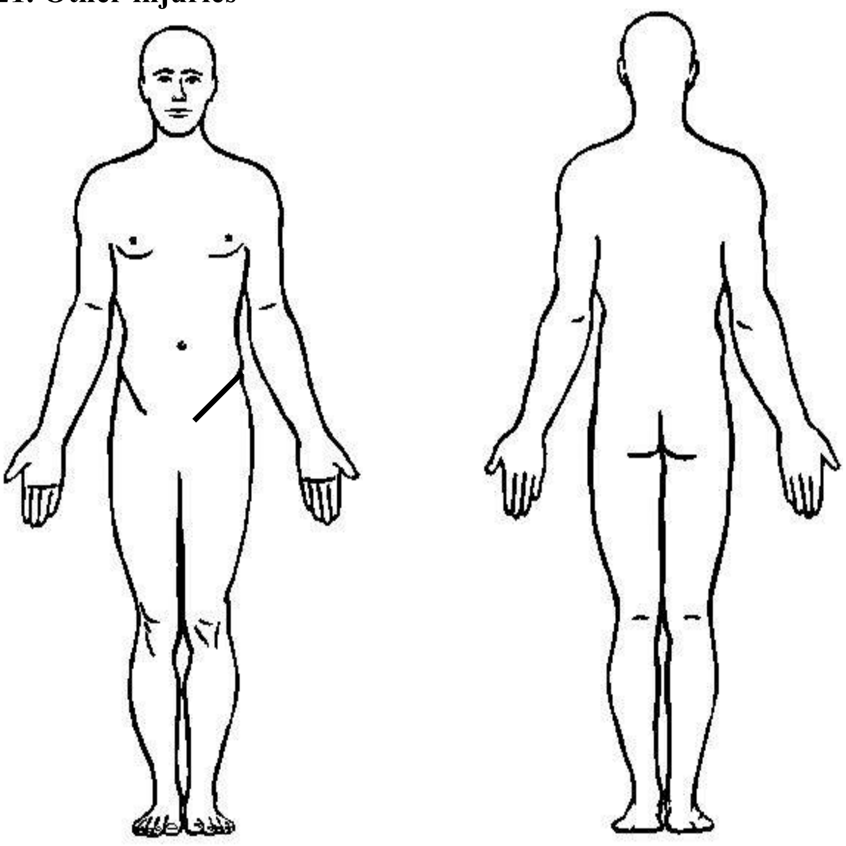

If >3 injuries use remarks

Mark each wound by number

1. ______________________________

Brief description of size and depth

2. ______________________________

Brief description of size and depth

3. ______________________________

Brief description of size and depth

Investigators signature ______________________ / ______ / ______

day month year

Validated by and date: ______________________

Preoperative Data

Patient no ☐

22. Preoperative haemoglobin

☐ g/dL ☐ Unknown

23. Preoperative transfusions

☐ None given ☐ Unknown

☐ Units of whole blood

☐ Units of packed red blood cells

☐ Units of Platelets

☐ Units of Plasma

24. Use of preoperative radiology

☐ No ☐ Unknown

☐ Computed Tomography (CT)

If CT was used, please specify findings

☐ Conventional radiology (X-ray)

If X-ray was used, please specify findings

Investigators signature ______________________ / / ______

day month year

Validated by and date: ______________________

Operative Data

Patient no [ ]

25. Time and date of procedure

[:] hours minutes [/ /] day month year [ ] Unknown

26. Duration of procedure

[:] hours minutes [ ] Unknown

27. Antibiotics prophylaxis

[ ] No [ ] Yes [ ] Unknown

If yes, please specify type, dose and duration

28. Primary procedure done

If lack of space use remarks

(If several procedures are relevant. Mark most relevant with S.)

Primary haemostasis (damage control) [ ] Yes [ ] No [ ] Unknown

Removal of foreign bodies [ ] Yes [ ] No [ ] Unknown

Raphi of bowel [ ] Yes [ ] No [ ] Unknown

If raphi, please specify site(s) and count(s)

Small intestinal resection with anastomosis [ ] Yes [ ] No [ ] Unknown

---

If anastomosis, please specify site(s) and technique(s)

Investigators signature ____________________ / /

day month year

Validated by and date: ____________________

Operative Data cont.

Patient no ☐

Primary procedure done cont.

Colon resection with anastomosis ☐ Yes ☐ No ☐ Unknown

If anastomosis, please specify site(s) and technique(s)

Stoma/Bowel deviation ☐ Yes ☐ No ☐ Unknown

If stoma(s), please specify site(s) and technique(s)

Vascular repair ☐ Yes ☐ No ☐ Unknown

If vascular repair(s), please specify location(s) and technique(s)

Splenectomy ☐ Yes ☐ No ☐ Unknown

Other ☐ Yes ☐ No ☐ Unknown

If other(s), please specify location(s) and procedure(s)

29. Perioperative complication ☐ Yes ☐ No ☐ Unknown

If yes, please specify type

Investigators signature ______________________ / /
day month year

Validated by and date: ______________________

Postoperative Data

Patient no [ ]

30. Postoperative antibiotic treatment

(not preoperative or prolonged prophylaxis)

[ ] No [ ] Unknown [ ] Yes

If yes, please specify type, doses and duration

31. Infected tissue

(If several, is possible, mark all with appropriate postop. day.)

[ ] No [ ] Unknown

Appendix 3 for definition

	Date (day / month / year)	
Affecting the skin and subcutaneous tissue	/ /	Date of diagnosis
Affecting the fascial and muscle layers	/ /	Date of diagnosis
Affecting abdominal cavity	/ /	Date of diagnosis
Sepsis	/ /	Date of diagnosis

Measures taken (E.g. intensive care, iv antibiotics, dialysis)

Unspecified infection / / Date for diagnosis

Please explain and measures taken

Investigators signature ______________________ / /

day month year

Validated by and date: ______________________

EMC Emergency Managment Center — Karolinska Institutet — LiU Linköping University

Postoperative Data cont.

Appendix 2 for definitions

Patient no [ ]

32. Other postoperative complication

[ ] No [ ] Unknown [/ /] Yes, date of complication

Type and measures taken

33. Need of postoperative intensive care

[ ] No [ ] Unknown [/ /] Yes, date of admission ICU

If yes, please specify reason(s) for intensive care

Discharged from Intensive Care Unit [/ /] Date

34. Postoperative transfusions

[ ] None given [ ] Unknown

[ ] Units of whole blood

[ ] Units of packed red blood cells

[ ] Units of Platelets

[ ] Units of Plasma

35. Use of postoperative radiology

[ ] No [ ] Unknown [ ] CT [ ] X-ray

If use of radiology, please specify findings

Investigators signature ______________________ / /

day month year

Validated by and date: ______________________

EMC Emergency Managment Center Karolinska Institutet LiU Linköping University

Postoperative Data cont.

Patient no ☐

36. Further surgery done

☐ No ☐ Unknown ☐ / / 1. Yes, date of surgery

1. ______________________________

If yes, please specify procedure done and if done because of complication(s) to primary procedure

____

____

☐ / / 2.Yes, date of surgery

2. ______________________________

If yes, please specify procedure done and if done because of complication(s) to primary procedure

____

____

☐ / / 3.Yes, date of surgery

3. ______________________________

If yes, please specify procedure done and if done because of complication(s) to primary procedure

____

____

Investigators signature ______________________________ / /

day month year

Validated by and date: ______________________________

Discharge Data

Patient no [ ]

37. Discharge

[ ] No [ ] Unknown

Date of discharge

/ /
day month year

Discharged Status

[ ] Hospital care complete

[ ] Left Against Medical Advice

[ ] Unknown

[ ] Transferred for further care

Transferred to where

Type of care planned

Investigators signature ________________ / /
day month year

Validated by and date: ________________

Follow-up Data

Patient no ☐

38. Follow-up planned ☐ No ☐ Yes ☐ Unknown

39. Follow-up scheduled at ____________________

day/month/year or days from discharge

40. Planned care provider for follow-up ☐ EH ☐ Any ☐ Unknown

41. Plan for follow-up ____________________

E.g. removal of sutures, change of dressing, further surgery etc.

---

42. Follow-up at EH was done on ☐ / / (day month year) ☐ Unknown ☐ Failed to come

43. Measures taken at follow-up ☐ As planned, according to question 41 ☐ Re-admitted ☐ Other ☐ Unknown

44. If other measures were taken ____________________

Please specify, also if related to previous procedures

Investigators signature ____________________ / /

day month year

Validated by and date: ____________________

EMC Emergency Managment Center سه نته ری چاره سه ری فریاکوزای Karolinska Institutet LINKÖPING UNIVERSITY

Follow-up Data cont.

Patient no [ ]

45. Further follow-up scheduled

[ ] Yes [ ] No [ ] Other plan [ ] Unknown

46. If further follow-up or other plan

____
If further follow-up or other, please specify plan/reason

47. If re-admitted at any follow-up

[/ /] Yes, date of re-admission [ ] No [ ] Unknown

____
Reason for re-admission and measures taken during this hospital stay

____

48. Date of discharge for re-admission

[/ /] day month year [ ] Unknown

49. Follow-up after re-admission

[ ] No [ ] Yes [ ] Unknown

50. Plan for follow-up after re-admission

____
Please, specify

Investigators signature ______________________________ / /

day month year

Validated by and date: ______________________________

EMC Emergency Managment Center Karolinska Institutet LINKÖPING UNIVERSITY

Exploratory laparotomy following penetrating abdominal injuries: cohort study from a referral hospital in Erbil, Kurdistan region in Iraq. Case Report Form (CRF), version 180302

Follow-up Data cont.

Patient no [ ]

51. Deceased

[ ] No [ ] Yes [ ] Unknown

Cause of death ______________________ [ ] Unknown

Date of death [/ /]

day month year

52. Remarks

____

____

____

____

____

____

____

____

____

Investigators signature ______________________ / /

day month year

Validated by and date: ______________________
